# The neural basis underlying impaired attentional control in problematic smartphone users

**DOI:** 10.1038/s41398-021-01246-5

**Published:** 2021-02-18

**Authors:** Jihye Choi, Hyun Cho, Jung-Seok Choi, In Young Choi, Ji-Won Chun, Dai-Jin Kim

**Affiliations:** 1 Department of Psychiatry, Seoul St. Mary’s Hospital, The Catholic University of Korea College of Medicine, Seoul, 06591 Republic of Korea; 2 Department of Psychiatry, SMG-SNU Boramae Medical Center, Seoul, 07061 Republic of Korea; 3 Department of Medical Informatics, The Catholic University of Korea College of Medicine, Seoul, 06591 Republic of Korea

**Keywords:** Neuroscience, Human behaviour

## Abstract

As a portable media device that enables ubiquitous access to friends and entertainment, smartphones are inextricably linked with our lives. Although there is growing concern about the detrimental effect of problematic smartphone use on attentional control, the underlying neural mechanisms of impaired attentional control in problematic smartphone users (PSU) has yet to be investigated. Using a modified cognitive conflict task, we examined behavioral performance in the presence of distracting words during functional magnetic resonance imaging in 33 PSU and 33 control participants (CON). Compared with the CON group, the PSU group demonstrated impaired performance that was accompanied by constantly enhanced but not differentiated activation in the frontoparietal regions across all conditions, regardless of distractor saliency. The inferior parietal lobule (IPL) activation in the PSU group, in particular, showed an association with performance deficits in the distractor conditions. Furthermore, the PSU group exhibited decreased functional connectivity of the right IPL with the right superior temporal gyrus of the ventral attention system in the attention-demanding condition relative to the easiest condition, which was associated with the severe dependence on smartphone use. Our findings suggest that greater distractibility in the PSU group during the attentional control task may be associated with inefficient recruitment of the ventral attention network involved in bottom-up attentional processing, as indicated by hyperactivation but less coherence within the network. The present study provides evidence for understanding the neural mechanisms underlying the impaired ability to keep attention from being oriented to task-irrelevant stimuli observed in PSU.

## Introduction

As portable media devices that enable ubiquitous access to the Internet, smartphones have become pervasive in our lives and transformed our ways of thinking, socializing, and entertaining. In a media-saturated environment, an individual with a smartphone can have immediate and easy access to friends, entertainment, and escapism^1^; thus, the use of a smartphone itself may act as a reward that leads to the risk of the addictive use of smartphones. In everyday lives, smartphone users sometimes express their growing concerns about their distractibility and habitual checking behavior with their smartphones^2^, which frequently interrupts their work and makes it difficult to focus on work.

The potent impact of smartphones on individuals’ cognitive function, such as attention, has been well established. For young adults, smartphones were shown to be more reinforcing than food^3^, and the mere presence^4^ and the awareness of the notifications^5^ of the smartphones were sufficient to distract their attention and worsen their cognitive performance. Individuals who tended to simultaneously use more than one type of media via smartphones were more susceptible to interference by distractor stimuli and failed to filter out the irrelevant information during attention-demanding tasks^6–9^. Moreover, it has been reported that frequent multitasking may be associated with bottom-up processing^10^ as well as a reduced ability to control attention with increased prefrontal activity^9^.

Despite the accumulating empirical reports and growing concerns, the neuroimaging evidence for altered attentional control in problematic smartphone users (PSU) remains surprisingly scarce. Furthermore, most of the abovementioned previous research have been studied in experimental conditions in which the smartphone was presented; thus, these studies could not illustrate the attentional impairment on the baseline level that the PSU may experience in their everyday lives when there is a need to focus on a particular primary task. Attentional control refers to an individual’s capacity to focus on task-relevant information while filtering out task-irrelevant information. It is well-known that attentional control involves a widespread network of frontal and parietal brain regions, consisting of parts of the dorsal and ventral frontoparietal components^11–13^. The dorsal network is assumed to be involved in the top-down voluntary orientation of attention for goal-relevant targets and comprises the superior parietal lobule (SPL) and frontal eye fields^12^. The ventral network is implicated in stimulus-driven attentional control, which is modulated by the detection of unexpected or infrequent stimuli that are behaviorally relevant^14,15^. The ventral network comprises the temporoparietal junction extending from the superior temporal gyrus (STG) to the inferior parietal lobule (IPL), and ventral frontal cortex (VFC), which is lateralized to the right hemisphere^11,16–18^. The parietal part of the ventral network was shown to be especially deactivated when subjects sustained attention and filtered irrelevant stimuli, preventing reorientation to unimportant information. Besides, a positron emission tomography study reported the involvement of prefrontal regions in maintaining attentional control during a conflict task^19^.

Here, we used functional magnetic resonance imaging (fMRI) to investigate the neural correlates of smartphone-related attentional control in groups of PSU and control subjects (CON) during the performance of a modified cognitive conflict task that contains the prominent features of the Stroop^20^ and Eriksen flanker^21^ tasks. The modified cognitive conflict task paradigm used color-word stimulus items for the target and distractors, and the mismatch of the printed color and meaning of the word is considered to produce the Stroop effect^20^. In addition, the target word in this task was flanked by the distracting stimuli that shared similar features with the target, and thus the task demanded the ability to focus and sustain attention to the target while inhibiting the responses to the distractors. The distracting words were designed to play a similar role to banner advertisements or irrelevant information that we may experience when we use smartphones. We expected to observe impaired performance in the presence of the distractors in the PSU group compared with the CON group since the PSU group may be susceptible to distractors and likely to make inappropriate responses. Moreover, the PSU group was expected to show concomitant altered ventral frontoparietal activation engaged in bottom-up processing and an association between this altered activation, particularly in the parietal region, and the behavioral performance. Furthermore, we expected that the PSU group, compared with the CON group, would exhibit weakened functional connectivity between regions of the ventral attention network in the more demanding condition relative to the easy condition, which might be associated with the severity of the smartphone dependency.

## Methods

### Participants

Sixty-six smartphone users in their 20s and 30s were included in the analysis. We divided the participants into two groups, PSU (*n* = 33; M/F = 15/18; mean age ± SD = 25.21 ± 5.54) and CON (*n* = 33; M/F = 15/18; mean age ± SD = 24.85 ± 4.49) groups, on the basis of the clinician-administered interview on smartphone usage patterns and the extent of the smartphone dependency referring to diagnostic criteria (e.g., preoccupation, tolerance, withdrawal, persistence, escape, problems, deception, displacement, and conflict) for Internet gaming disorder suggested in Section 3 of the fifth edition of the Diagnostic and Statistical Manual of Mental Disorders (DSM-5) in 2013^22^. There was no participant with substance or alcohol use disorder according to the clinician-administered the Mini-International Neuropsychiatric Interview. We assessed the severity of smartphone dependency using the self-reported smartphone addiction proneness scale (K-SAPS)^23^, developed by the Korean National Information Society in 2011. The K-SAPS is composed of 15 items and contains four subdomains, which represent the distinct characteristics of addictive smartphone use: disturbance of adaptive function, virtual life orientation, withdrawal, and tolerance. The reliability of the K-SAPS is acceptable (Cronbach’s alpha = 0.814). The participant was classified in a high-risk group if the score exceeded 44 and in an at-risk group if the score was 40–43. The PSU group had significantly higher K-SAPS scores than the CON group (PSU group: 39.76 ± 6.75, CON group: 26.76 ± 5.76; *t*_(64)_ = 8.415, *p* < 0.001). All participants graduated from high school, and their intelligence quotient (IQ) was above 85. The two groups did not significantly differ in IQ (PSU group: 106.21 ± 8.82, CON group: 109.82 ± 7.00; *t*_(64)_ = −1.840, *p* = 0.070). They had a normal or corrected-to-normal vision and no history of any major medical disorders, neurological disorders, or head injuries. They gave written informed consent approved by the Institutional Review Board of Seoul St. Mary’s Hospital in South Korea, by which all experimental protocols were approved.

### Experimental paradigm

**Fig. 1:**
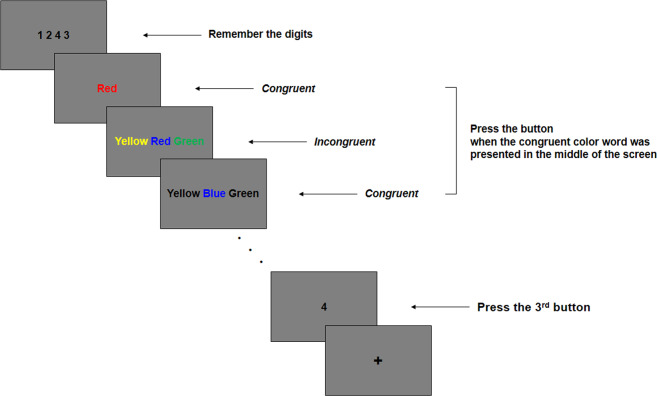
Illustration of the experimental task. We modified the traditional Stroop color-word task and Eriksen flanker task to examine the effect of distractor interference on attentional control. The target word was presented in the middle of the screen, and the participants were instructed to press a button when the printed color and meaning of the target word were congruent, regardless of the distractor words flanked on either side of the target word. Furthermore, it consisted of a study phase in which four digits (ranging from 1 to 4) to be remembered were presented for 1.5 s with a 100-ms blank screen before each experimental block started and a test phase in which the participant was asked to press the button corresponding to the order of the presented number previously shown in the study phase.

Figure 1 illustrates the modified cognitive conflict task paradigm. We modified the traditional Stroop color-word task^20^ and Eriksen flanker task^21^ to examine the effect of distractor interference on attentional control. The target word was presented in the middle of the screen, and the participants were instructed to press a button when the printed color and meaning of the target word were congruent, regardless of the distractor words flanked on either side of the target word. Since each trial was presented briefly, i.e., 1000 ms with 100-ms blank screen, the participants were asked to respond as accurately and quickly as possible. We differentiated the distracting stimuli in each experimental condition to assess the effect of varying the attentional workload across conditions. Therefore, the paradigm incorporated four conditions: None, 1-distractor (1D), 2-distractor (2D), and Black-distractor (BD) conditions. In the None condition, only the target color word, with no distractor, was presented, while the color of the distractor words was black in the BD condition. In the 1D condition, the different color distractor words were added on either side of the target word, so three colors were presented in each trial. In the 2D condition, one of the distractor color words was printed the same color as the target word’s printed color. Each experimental block consisted of 15 trials for 16.5 s, among which 2–3 incongruent trials were included, and a fixation cross was presented for 20 s between the experimental condition blocks. Furthermore, we added a recognition task in which the participants were required to remember the four digits to be answered while performing the following cognitive conflict task. It consisted of a study phase in which four digits (ranging from 1 to 4) to be remembered were presented for 1.5 s with a 100-ms blank screen before each experimental block started and a test phase in which the participant was asked to press the button corresponding to the order of the presented number previously shown in the study phase. Therefore, the participants were expected to maintain their attention during the whole experimental block and not to get distracted by either the recognition task or the cognitive conflict task. During fMRI scanning, two sessions were conducted, each of which consisted of eight blocks (four conditions × twice), resulting in a total duration of 5 min and 30 s. Prior to the task, participants practiced two blocks of the task to familiarize them with the protocol of the task.

### MRI data acquisition

MRI data were acquired using a 3 Tesla Siemens MAGNETOM Trio scanner (Siemens, Erlangen, Germany). Functional data were acquired using an echo-planar imaging sequence with the following parameters: repetition time (TR) = 2000 ms, echo time (TE) = 30 ms, matrix = 64 × 64, the field of view (FOV) = 220 mm, flip angle = 90°, 32 interleaved slices with a thickness of 5.0 mm and without gaps, and 162 volumes with a total scan time of 5 min and 30 s. For normalization of the functional MRI data, T1-weighted magnetization-prepared rapid gradient-echo images were collected with the following parameters: TR = 2300 ms, TE = 2.52 ms, matrix = 256 × 256, FOV = 256 mm, flip angle = 9°, voxel size = 1 × 1 × 1 mm, 192 slices with a thickness of 1.0 mm and without gaps, and scan duration = 5 min 21 s.

### MRI preprocessing and first-level analysis

Image data were preprocessed using the CONN functional connectivity toolbox (version 17.f; https://www.nitrc.org/projects/conn)^24^ with its default preprocessing pipeline, in conjunction with Statistical Parameter Mapping 12 (SPM12, Wellcome Department of Imaging Neuroscience, London, UK), implemented in MATLAB 2014a (Mathworks, Sherborn, MA, USA). After discarding the first five functional image volumes from the dummy scan in each session, the remaining 157 images were realigned to adjust for head movements, unwarped, slice-time corrected, coregistered to each participant’s T1 image, normalized to Montreal Neurological Institute (MNI) coordinate space, and spatially smoothed with a Gaussian kernel of 8-mm full-width at half-maximum.

For individual analyses, a general linear model framework was performed to model the blood-oxygen-level-dependent (BOLD) signal convolved with the canonical hemodynamic response function using SPM12. The design matrix contained the experimental conditions of None, 1D, 2D, and BD conditions as separate regressors of interest and all other conditions of the recognition task and fixation cross and head movement parameters as regressors of no interest. Data were high-pass filtered at 128 s.

### Brain activation analysis

We conducted a second-level random-effects group analysis using a flexible factorial design to examine the brain regions that showed a difference between groups and conditions (FWE-corrected *p* < 0.05, *k* > 100). To account for the between-group differential activation in each condition, *post hoc* two-sample *t* tests were performed under the flexbile factorial design (FWE-corrected *p* < 0.05, *k* > 100). Next, we chose the right IPL and middle frontal gyrus (MFG), known as hubs in the ventral attentional network, to examine whether the activation pattern was different based on the saliency of the distractors. We generated 10-mm spherical masks centered on the peak voxel of the right IPL and MFG, identified as increased activation in the PSU group compared with the CON group across all conditions, that were restricted by the corresponding regions from the automated anatomical labeling atlas (IPL MNI coordinates: *x* = 38, *y* = −60, *z* = 50; MFG MNI coordinates: *x* = 48, *y* = 14, *z* = 46). Then, we extracted the percent BOLD signal change values of these two regions from each participant in each experimental condition using MarsBaR version 0.41 (http://marsbar.sourceforge.net). For simplicity of further correlation analyses, we converted the behavioral variables, i.e., the error rate (ER) and reaction time (RT), into a single measure, namely, the inverse efficiency score (IES), by dividing the mean correct RTs by the accuracy (1-ER) for each condition and in each participant. The IES represents an index of overall performance that accounts for potential speed-accuracy trade-offs, and higher IES values indicate worse overall performance^25^. Correlation analyses were conducted using IBM SPSS Statistics for Windows, version 20.0 (IBM SPSS, Armonk, NY, USA). Bonferroni corrections for multiple comparisons were made (*p* < 0.0125; 0.05/4 tests).

### Functional connectivity analysis

We explored the brain regions that were functionally connected in terms of the condition-dependent activity of the right IPL using a generalized psychophysiological interaction (gPPI) analysis^26^ implemented in the CONN functional connectivity toolbox^24^. Generalized PPI enables simultaneous evaluation of the task-modulated connectivity from all conditions in the estimation model. Here, we examined the distractor-modulation effects on the functional connectivity of the right IPL to other brain regions (height threshold uncorrected *p* < 0.001 with cluster-size FWE-corrected *p* < 0.05). To test whether altered IPL functional connectivity was related to the severity of the problematic smartphone use, we extracted the PPI parameters from the cluster that was significantly connected with the right IPL shown in the specific condition relative to the None condition and performed a Pearson’s correlation analysis between the PPI parameters and the K-SAPS scores in each group (two-tailed *p* < 0.05).

## Results

### Behavioral results

A two (group) by four (distractor condition) repeated measures ANOVA with Huynh–Feldt correction on the ERs yielded significant main effects of group (*F*_(1,64)_ = 16.62, *p* < 0.001) and distractor condition (*F*_(2.637, 192)_ = 32.307, *p* < 0.001). There was a trend for an interaction (*F*_(2.637, 192)_ = 2.609, *p* = 0.061).

Similar to the results with the ERs, RT analyses revealed significant main effects of group (*F*_(1, 64)_ = 15.242, *p* < 0.001) and distractor condition (*F*_(2.392, 192)_ = 24.120, *p* < 0.001), but there was no significant interaction between group and condition (*F*_(2.392, 192)_ = 1.084, *p* = 0.349).

Table 1 presents the behavioral performance of both groups. The participants showed more accurate and faster performance in the None condition compared with the other distractor conditions, i.e., 1D, 2D, and BD conditions, indicating the effect of distractor interference on performance on the cognitive conflict task. A follow-up two-sample *t* test revealed that the PSU group responded less accurately and more slowly in all conditions than the CON group (Table 1). Interestingly, even though only the target word was presented in the None condition, the PSU group exhibited significantly impaired task performance compared with the CON group. Accordingly, overall performance, as indicated by the inverse efficiency score (IES), confirmed that the PSU group performed worse in the task than the CON group in each condition.

**Table 1:** Behavioral performance.

	PSU group	CON group	*t*	*p* Value
*Error rate (%)*				
None	0.012 ± 0.019	0.001 ± 0.005	3.141	0.003
1D	0.066 ± 0.064	0.027 ± 0.040	2.917	0.005
2D	0.075 ± 0.061	0.038 ± 0.041	2.932	0.005
BD	0.075 ± 0.042	0.038 ± 0.036	3.839	<0.001
*Reaction time (ms)*				
None	526.75 ± 43.36	484.91 ± 35.21	4.304	<0.001
1D	553.79 ± 46.68	511.02 ± 39.24	4.029	<0.001
2D	545.21 ± 53.35	513.32 ± 36.88	2.825	0.006
BD	537.21 ± 53.35	500.86 ± 33.39	3.318	0.002
*IES*				
None	533.56 ± 49.96	485.53 ± 35.82	4.488	<0.001
1D	595.37 ± 66.66	526.47 ± 48.59	4.799	<0.001
2D	592.34 ± 72.01	534.60 ± 48.31	3.825	<0.001
BD	582.95 ± 72.22	521.85 ± 45.02	4.125	<0.001

Abbreviations: *CON* control, *IES* inverse efficiency score, *PSU* problematic smartphone user.

In the recognition task, we found significant main effects of group (*F*_(1, 64)_ = 7.616, *p* = 0.008) and condition (*F*_(2.780, 192)_ = 4.265, *p* = 0.008) on accuracy but no interaction (*F*_(2.780, 192)_ = 0.547, *p* = 0.637). However, with RTs, we found no main effects of group (*F*_(1, 64)_ = 0.117, *p* = 0.733) and condition (*F*_(3, 192)_ = 0.404, *p* = 0.750) and interaction (*F*_(3, 192)_ = 0.977, *p* = 0.405).

### fMRI results

#### Brain activation

The whole-brain analyses revealed a significant main effect of condition in the bilateral occipital cortex extending to the SPL (Table 2). The subsequent *t* tests demonstrated that the participants in both groups showed increased activation in the 1D condition but decreased activation in the None condition.

**Table 2:** Brain regions showing activation differences between groups in each condition.

Contrast/region	MNI coordinates			*z*-value	Cluster size
	*x*	*y*	*z*		
*Condition effects*					
R. Lingual gyrus	16	−78	−8	>8	585
L. Calcarine cortex	−8	−88	−2	>8	542
R. Calcarine cortex	8	−88	0	>8	322
L. Precuneus	−18	−66	46	6.13	281
*Group effects*					
None					
*PSU* < *CON*					
L. Middle occipital lobe	−18	−94	14	>8	187
*PSU* > *CON*					
R. Angular gyrus/inferior parietal lobule	38	−60	50	>8	341
R. Fusiform gyrus	30	−80	−10	7.51	139
L. Supplementary motor area	−2	6	60	7.49	240
R. Supramarginal gyrus	56	−26	44	6.98	276
R. Middle frontal gyrus	46	12	48	6.74	317
B. Paracentral gyrus	0	−34	56	6.58	332
L. Inferior parietal lobule	−28	−48	46	6.55	668
L. Superior parietal lobule	−24	−72	52	6.26	177
L. Precentral gyrus	−18	−16	72	6.20	474
L. Precentral gyrus	−38	−8	44	5.74	147
L. Inferior frontal gyrus	−40	12	26	5.67	198
L. Middle cingulate gyrus	−10	−26	44	5.62	127
L. Precuneus	−2	−54	42	5.1	163
1D					
*PSU* < *CON*					
L. Middle occipital lobe	−18	−94	16	>8	682
*PSU* > *CON*					
R. Angular gyrus/inferior parietal lobule	38	−60	50	>8	527
R. Middle frontal gyrus	50	22	36	6.08	210
R. Supramarginal gyrus	54	−28	42	5.44	103
2D					
*PSU* < *CON*					
L. Middle occipital lobe	−18	−94	14	>8	406
*PSU* > *CON*					
R. Angular gyrus/inferior parietal lobule	38	−60	50	7.11	158
BD					
*PSU* < *CON*					
L. Middle occipital lobe	−18	−94	14	>8	863
*PSU* > *CON*					
R. Angular gyrus/inferior parietal lobule	38	−60	50	5.90	155^†^

Abbreviations: *B* bilateral, *CON* control, *L* left, *PSU* problematic smartphone user, *R* right.

^*^Significant at FWE-corrected *p* < 0.05, *k* > 100.

^†^Significant at FWE-corrected *p* < 0.05, *k* > 100 after small volume correction.

**Fig. 2:**
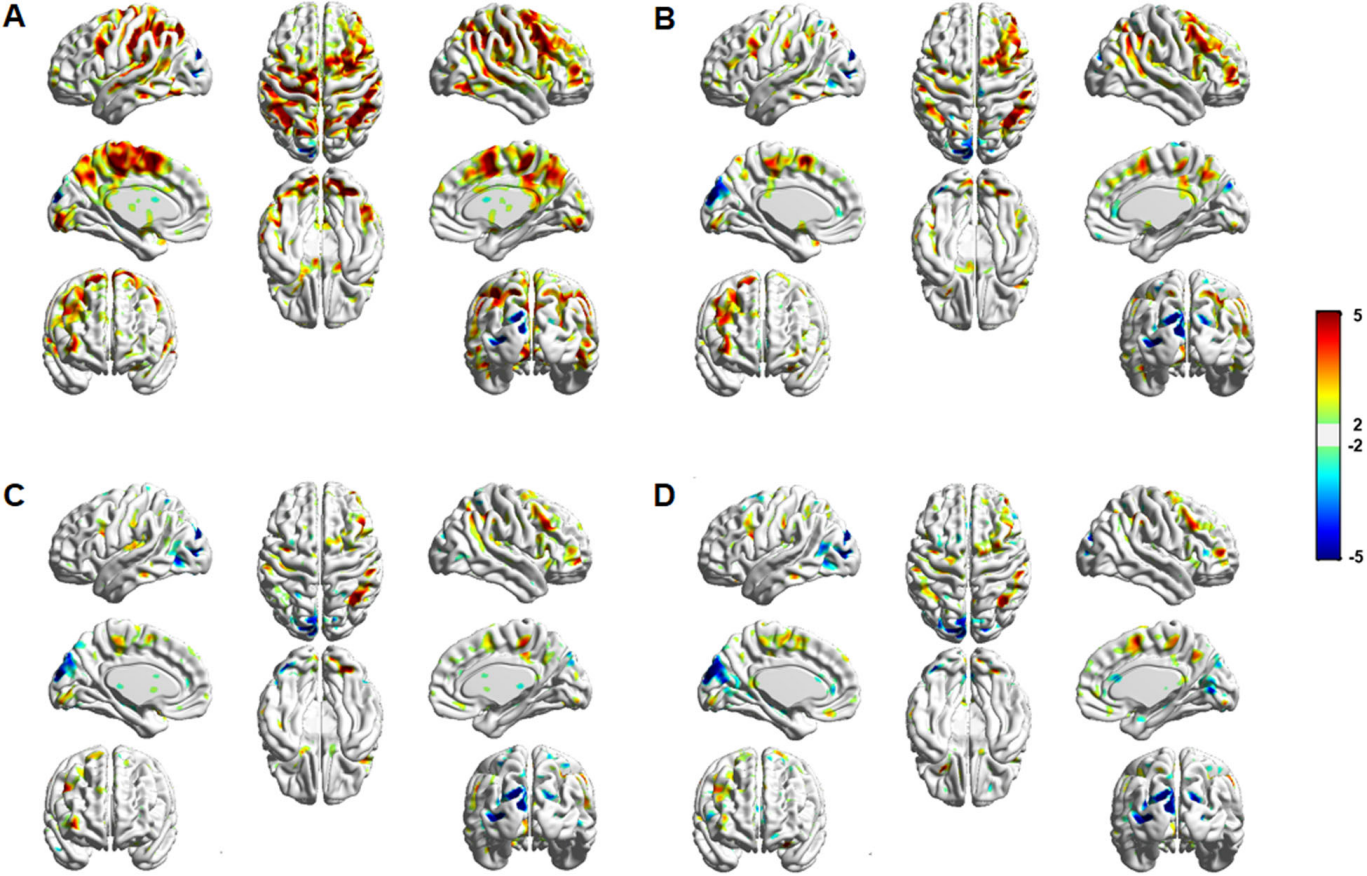
Brain activation differences between the PSU group and the CON group in each condition during the modified cognitive conflict task. **A** None, **B** 1D, **C** 2D, and **D** BD conditions. Red represents the brain areas that showed greater activation in the PSU group than in the CON group. The right side of the image corresponds to the right hemisphere of the brain.

A significant main effect of group was observed in multiple frontoparietal and occipital regions. Figure 2 presents the results of *post hoc* group comparison analyses for each condition separately. In all conditions, the PSU group, compared with the CON group, demonstrated greater activation in the frontoparietal areas, including the right IPL and MFG, while exhibiting decreased activation in the left middle occipital lobe. There was no brain region showing a condition and group interaction effect.

**Fig. 3:**
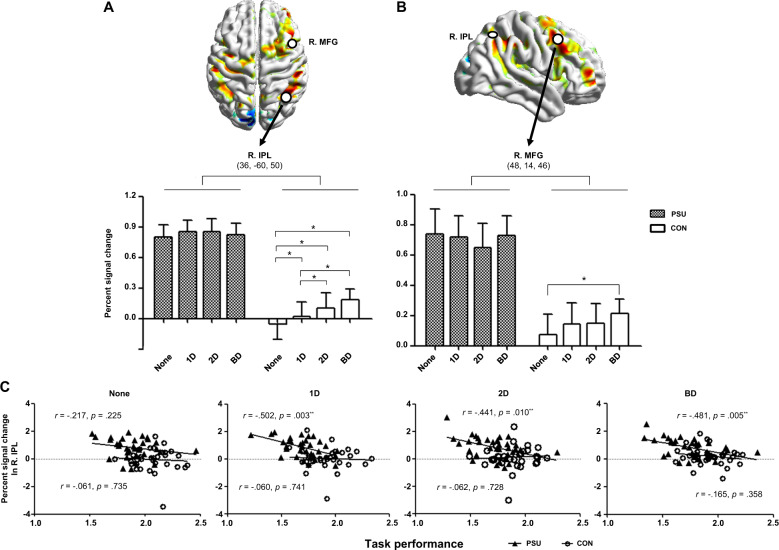
The brain activation patterns and the correlation between the activation and task performance. The activation patterns in the **A** right IPL and **B** MFG in each condition that was different between the two groups. ^*^Significant at *p* < 0.05. **C** The correlation between the right IPL activation and the task performance in each condition. The task performance was presented as the inverse score of the inverse efficiency score (IES) for visualization. ^**^Significant at *p* < 0.0125, corrected.

In addition, different patterns of activation in the right IPL and MFG were observed between the groups (Fig. 3A, B). The right IPL and MFG activation in the PSU group did not differ across conditions, while the CON group exhibited distinct activation in a distractor saliency load-dependent manner. The enhanced but not differentiated IPL activation in the PSU group was negatively correlated with the performance in the distractor conditions, which was not observed in the CON group (Fig. 3C). The results survived the Bonferroni correction for multiple comparisons (*p* < 0.0125). However, correlations between the MFG activation and the performance in each condition were not significant in either group (None: PSU group, *r* = −0.063, *p* = 0.73; CON group, *r* = −0.023, *p* = 0.90; 1D: PSU group, *r* = 0.107, *p* = 0.55; CON group, *r* = 0.134, *p* = 0.46; 2D: PSU group, *r* = 0.172, *p* = 0.34; CON group, *r* = 0.087, *p* = 0.63; BD: PSU group, *r* = 0.017, *p* = 0.92; CON group, *r* = 0.183, *p* = 0.31).

#### Functional connectivity

**Fig. 4:**
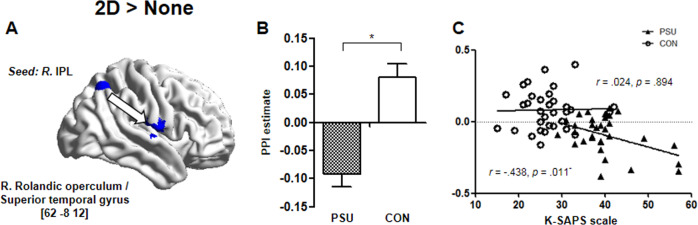
The effect of problematic smartphone use on the functional connectivity of the right IPL in the presence of distracting stimuli during the behavioral performance. **A**, **B** Compared with the CON group, the PSU group showed decreased functional connectivity between the right IPL and rolandic operculum/STG during the 2D condition relative to the None condition. ^*^Significant at *p* < 0.05. **C** The PSU group demonstrated a negative association between the strength of this functional connectivity and the severity of the smartphone dependency measured by the K-SAPS scale. ^*^Significant at *p* < 0.05. Error bars indicate SEM. CON control group, K-SAPS smartphone addiction proneness scale, developed by the Korean National Information Society in 2011, IPL inferior parietal lobule, PPI psychophysiological interaction, PSU problematic smartphone user group.

The gPPI analysis revealed that compared with the CON group, the PSU group displayed reduced functional connectivity of the right IPL with the right rolandic operculum extending to the STG during the 2D condition relative to the None condition (Fig. 4A, B) (MNI coordinates: *x* = 64, *y* = −8, *z* = 12; *k* = 220, height threshold uncorrected *p* < 0.001 with cluster-size FWE-corrected *p* = 0.007). In addition, this functional connectivity strength was negatively correlated with the self-reported severity of the smartphone dependence in the PSU but not in the individuals in the CON group (Fig. 4C) (PSU group: *r* = −0.438, *p* = 0.011; CON group: *r* = 0.024, *p* = 0.894). However, there was no brain region showing increased functional connectivity in the PSU group compared with the CON group.

## Discussion

This is the first study, to our knowledge, to investigate the neural mechanisms underlying the effect of distractor interference on attentional control in PSU. The PSU group exhibited performance decrements and enhanced activation in widespread frontoparietal brain areas during all conditions compared with the CON group. It is also confirmed that these specific areas were recruited for attentional control, based on the finding that as the distractor saliency, i.e., attentional workload, increased, the difference in the frontoparietal activations between the two groups tended to decrease. However, the activation patterns of the ventral frontoparietal regions were different between the two groups, illustrating that the PSU group appeared to inefficiently recruit the neural resources of the attention network. The IPL activation in the PSU group, in particular, showed an association with performance deficits in the distractor conditions, with the exception of the None condition. Furthermore, compared with the CON group, the PSU group showed weakened functional connectivity within the right ventral attention network during the 2D condition relative to the None condition, which was associated with the severe dependence on smartphone use. These findings suggest that greater distractibility in the PSU group during the attentional control task may be associated with less efficient recruitment of the ventral attention network.

The current cognitive conflict task paradigm demands the capacity to sustain attention on the target word and inhibit voluntary responses to the distracting stimuli that shared the common features with the target word. Both groups performed better in the None condition than in the other distractor conditions, which confirmed the interference effect of the distractors on behavioral performance. However, slower and less accurate performance across all conditions in the PSU group implicates smartphone-related impairments in attentional control. This finding provides novel evidence that PSU may have lower baseline levels of attention in a context without smartphones because previous studies reporting the interference effects of smartphones on attention have been conducted in the presence of smartphones during task performance^4–9^.

The whole-brain analyses revealed that greater recruitment of the right IPL and MFG during the entire task was observed in the PSU group than in the CON group. This may demonstrate that the effect of problematic smartphone use on attentional control is more associated with the ventral attention system. The ventral attention system, comprising the right IPL and VFC (i.e., MFG and inferior frontal gyrus), is involved in stimulus-driven attentional control, especially when the unexpected stimuli are behaviorally relevant or share similar features with a target^12,17^. Because smartphones are salient in the environment and closely related to users’ personal lives, the constant interaction with smartphones is likely to draw the orientation of users’ attention and occupy attentional resources^27^. Therefore, PSU may frequently experience being distracted by their smartphones in everyday life, which would affect some brain areas that are related to stimulus-driven attentional control. Moreover, deactivation of the right IPL prevents an inappropriate response to irrelevant stimuli, indicating the essential role of this region in filtering unimportant information^12,17^. Thus, the failure to suppress the right IPL activation in the PSU group may have contributed to the increased sensitivity to the distractors, thus interfering with appropriate responses to the target.

In this study, the between-group difference in brain activation was attenuated as the attentional workload increased. This finding may illustrate that performing in distracting conditions was taxing for both groups. However, the different activation patterns of the ventral frontoparietal areas, such as the IPL and MFG, indicated smartphone-related alterations with respect to neural efficiency. Since human attention is a limited cognitive resource that employs the selection of task-relevant information from environmental stimuli at the expense of ongoing neural activity, how efficiently and appropriately individual recruits neural resources could be indicative of better cognitive performance^28^. In terms of neural efficiency, although increased brain activation reflects increased recruitment of the neural resources, equal or superior performance and concomitant less activation have been deemed indicative of more efficient recruitment of cognitive reserve^29^. Accordingly, we can assume that the PSU group inefficiently recruited the neural resources for attentional control processing on the basis of their performance impairment and constantly enhanced activation during all conditions. In addition, they exhibited that greater IPL activation was associated with worse performance. On the other hand, the CON group appeared to reflect higher neural efficiency, which was manifested as the superior performance with less activation but showed changes in activity in a distractor load-dependent manner.

It has been suggested that when the resources of the brain areas specialized for the task-relevant function are exhausted, the brain activation would spill over to additional areas less specialized for the same function to compensate for the increased functional demands of a task^30^. Here, compared with the CON group, the PSU group activated additional brain areas such as the SPL and medial frontal areas in the None condition relative to the other distraction conditions. These additional brain areas are also thought to be important for attentional control: the SPL and precuneus are considered key regions consisting of the dorsal frontoparietal attention network engaged in top-down processing, whereas the medial frontal areas have been reported to be involved in the detection of response selection^31,32^ and inhibition^33,34^ as well as distractor susceptibility^35^. Accordingly, the PSU group may need to engage compensatory mechanisms to meet task demands, even at lower attentional workload levels, as the finding showed the PSU group activated additional brain areas in the None condition compared with the CON group. Despite the engagement of additional areas, the PSU group displayed performance deficits in the None condition, which did not correlate with the brain activation patterns, either. This may demonstrate the PSU group’s impaired ability to efficiently utilize the corresponding resources to the task demands.

Our gPPI connectivity analysis revealed that the right IPL in the PSU group, compared with that in the CON group, had weakened connectivity with the right rolandic operculum extending to the STG during the 2D condition relative to the None condition. In addition, this connectivity was negatively correlated with the severity of smartphone dependence in the PSU group but not in the CON group. Since the rolandic operculum primarily includes the secondary somatosensory cortex^36^, the involvement of the rolandic operculum in attention has been previously reported with regard to the somatosensory modality^37,38^ and reorientation during emotional processing^39,40^. Meanwhile, research on spatial neglect may provide another explanation. Spatial neglect is a syndrome characterized by an inability to orient and respond to contralesional stimuli despite normal visual perception. The primary regions damaged in neglect contain the right rolandic operculum and STG as well as the right IPL^41,42^, which constitute the ventral attention network^43^. This anatomical and symptomatic similarity between the neglected patients and the current participants who have normal vision but showed impaired attentional control may suggest the plausible role of the right rolandic operculum/STG in the reorientation of attention toward unexpected stimuli. Furthermore, reduced coherence between these regions of the ventral attention system in the PSU group during more difficult 2D conditions was associated with more severe dependency on smartphone use. These gPPI findings may provide important evidence for less efficient neural communication within the ventral attention network related to problematic smartphone use.

Several limitations to the present study need to be considered. First, the results of our cross-sectional study should be interpreted with caution. We cannot determine whether the problematic smartphone use causes deficits in attentional control or people who already experience attentional deficits are more likely to become addicted to smartphones. A longitudinal study is needed to elucidate the causality of this association. Second, although the negative effect of smartphone technology on cognition has drawn the attention of the public and researchers, the fundamental concept of smartphone addiction, or problematic smartphone use, is still unclear and has been broadly used. In addition, the potential variables that trigger problematic smartphone use, such as the main purposes of smartphone use, would be worth consideration. Specifically, the effect of distractor interference on the brain and performance might be distinguished between individuals who primarily use smartphones for social network services and those who use them for games. Next, in the future, it would need to be considered measuring other scales related to addiction such as the Alcohol Use Disorders Identification Test to control for other addictive behaviors, although we confirmed that there was no participant with substance or alcohol use disorder in this present study. Also, it would be helpful to use the scales that assess the daily experiences of inattention associated with smartphone use to study the smartphone-related attention problems^44^. Lastly, even though we demonstrated the inefficient involvement of the frontoparietal attention system in attentional control related to problematic smartphone use, we could not separately analyze the performance during the recognition task in the neuroimaging analyses. In a further study using the event-related design, it would provide a better opportunity to explore the distractor interference effects on brain activation associated with correct vs. error trials of the recognition task.

The present findings make a valuable contribution to the field of research on the effect of smartphones on cognition since we provide the first neuroimaging evidence to account for the difficulty in attentional control that has been empirically reported in PSU. In conclusion, the PSU group demonstrated a cognitive conflict task performance decrement that was accompanied by constantly increased activation in the ventral frontoparietal regions during the conditions in which only the target word was presented as well as the conditions in which distracting stimuli were also presented. The finding of the negative association between the right IPL activation and behavioral performance in the PSU group during the distracting conditions may indicate that altered right IPL function may reflect their failure to filter unimportant information. Furthermore, the PSU group was found to have weakened connectivity within the ventral attention network in the more attention-demanding condition (2D condition) relative to the easiest None condition. In addition, weakened connectivity was shown to correlate with more severe dependence on smartphone use. These findings suggest that during the performance of the cognitively demanding task in the presence of distractors, greater distractibility in the PSU group during the attentional control task may be associated with less efficient recruitment of the ventral attention network involved in bottom-up attentional processing. The present study may provide evidence for an altered neural mechanism underlying the impaired ability to keep attention from being oriented to task-irrelevant stimuli observed in PSU.
